# *Remote* blood pressure monitoring in high risk pregnancy — study protocol for a randomised *control*led trial (REMOTE CONTROL trial)

**DOI:** 10.1186/s13063-023-07321-0

**Published:** 2023-05-17

**Authors:** Theepika Rajkumar, Jill Freyne, Marlien Varnfield, Kenny Lawson, Kaley Butten, Renuka Shanmugalingam, Annemarie Hennessy, Angela Makris

**Affiliations:** 1 School of Medicine, Western Sydney University, Penrith, NSW Australia; 2 Department of Medicine, Campbelltown Hospital, South Western Sydney Local Health District, Campbelltown, NSW Australia; 3 Australian E-Health Research Centre, Health and Biosecurity, CSIRO, Brisbane, QLD Australia; 4 Translational Health Research Institute, Western Sydney University, Penrith, NSW Australia; 5 Department of Renal Medicine, Liverpool Hospital, Liverpool, NSW Australia; 6 University of New South Wales, Kensington, NSW Australia

**Keywords:** Preeclampsia, Hypertensive disorders of pregnancy, Gestational hypertension, Blood pressure, Remote monitoring, Home blood pressure monitoring, Self-monitoring, High-risk pregnancy, Randomised controlled trial, Mobile health

## Abstract

**Background:**

Pregnant women at high risk for developing a hypertensive disorder of pregnancy require frequent antenatal assessments, especially of their blood pressure. This expends significant resources for both the patient and healthcare system. An alternative to in-clinic assessments is a remote blood pressure monitoring strategy, in which patients self-record their blood pressure at home using a validated blood pressure machine. This has the potential to be cost-effective, increase patient satisfaction, and reduce outpatient visits, and has had widespread uptake recently given the increased need for remote care during the ongoing COVID-19 pandemic. However robust evidence supporting this approach over a traditional face-to-face approach is lacking, and the impact on maternal and foetal outcomes has not yet been reported. Thus, there is an urgent need to assess the efficacy of remote monitoring in pregnant women at high risk of developing a hypertensive disorder of pregnancy.

**Methods:**

The REMOTE CONTROL trial is a pragmatic, unblinded, randomised controlled trial, which aims to compare remote blood pressure monitoring in high-risk pregnant women with conventional face-to-face clinic monitoring, in a 1:1 allocation ratio. The study will recruit patients across 3 metropolitan Australian teaching hospitals and will evaluate the safety, cost-effectiveness, impact on healthcare utilisation and end-user satisfaction of remote blood pressure monitoring.

**Discussion:**

Remote blood pressure monitoring is garnering interest worldwide and has been increasingly implemented following the COVID-19 pandemic. However, robust data regarding its safety for maternofoetal outcomes is lacking. The REMOTE CONTROL trial is amongst the first randomised controlled trials currently underway, powered to evaluate maternal and foetal outcomes. If proven to be as safe as conventional clinic monitoring, major potential benefits include reducing clinic visits, waiting times, travel costs, and improving delivery of care to vulnerable populations in rural and remote communities.

**Trial registration:**

The trial has been prospectively registered with the Australian and New Zealand Clinical Trials Registry (ACTRN12620001049965p, on October 11th, 2020).

## Background

Hypertensive disorders of pregnancy (HDP), and particularly preeclampsia contribute towards significant short- and long-term maternal morbidity and increase foetal morbidity and mortality 5–sixfold [1]. Women with chronic hypertension, kidney disease, preexisting diabetes mellitus, a previous HDP particularly preeclampsia, and autoimmune disease [2, 3] are considered high risk for developing preeclampsia. A recent study revealed the rate of HDP in this high-risk population was up to 33.8%, with an associated high rate of adverse foetal outcomes (intrauterine growth restriction or preterm delivery) between 15 and 17% [4]. The long-term maternal effects of preeclampsia are also increasingly recognised as a lifetime increased risk of chronic hypertension, coronary artery disease, renal disease and stroke [3, 5–7].

The United Kingdom (UK) National Institute for Health and Care Excellence (NICE) guidelines recommend that women at high risk be identified before week 12 of gestation, commence low-dose aspirin, and undertake more frequent blood pressure (BP) measurements than standard antenatal care [2, 8]. Consequently, this leads to additional outpatient appointments throughout their pregnancy [9], which expends significant resources at the level of both the patient and healthcare system. Frequent monitoring can be a source of anxiety for women and their families, is demanding for patients in terms of time, transport costs and work absence, and has significant service implications for healthcare providers [10].

In recent years, there has been an increasing focus on mobile health (mHealth) technologies, such as mobile phones, patient monitoring devices, and other wireless devices, to make healthcare delivery more efficient [11]. Compared to traditional methods of disease surveillance, mHealth has been found to have improved accuracy, reductions in time and cost, and improved data quality [11]. As such, remote BP monitoring, in which patients monitor and record their own BP using a validated machine, with instructions from a healthcare professional on the frequency of monitoring, has garnered increasing interest [10]. Additionally, the COVID-19 pandemic drastically limited social movement, and therefore initiated widespread uptake of telehealth services to reduce dependence on hospital‐based care, and remote BP monitoring was often required [12].

In the non-pregnant population, remote BP monitoring has been shown to provide a better estimate of underlying BP and long-term outcomes [13, 14]. In the pregnant population, a systematic review of clinical practice guidelines for hypertensive disorders of pregnancy found that most international guidelines recommended remote BP monitoring for hypertension control [15], yet robust evidence for this is lacking.

Studies exploring the use of remote BP monitoring in the antepartum have shown that remote BP monitoring is feasible in pregnant women [16–20], and may even be favourable to conventional clinic monitoring [21–24]. There were less outpatient antenatal visits and day assessment unit attendances [10, 17, 22, 25, 26] when compared to conventional care. There is also a potential beneficial economic impact to the healthcare system, which facilitates the widespread adoption of remote monitoring [27, 28]. No individual study was powered for maternal, foetal and neonatal outcomes as primary outcomes [10, 16, 20, 25–27]. A meta-analysis however found remote BP monitoring was associated with reduced odds of labour induction and prenatal hospital admissions, with no significant differences in foetal outcomes [29]. Similar findings are revealed in postpartum studies, with good feasibility, acceptability [30–34], and fewer hypertension-related hospital admissions [30, 33, 35]. Significant heterogeneity exists amongst these studies [17, 21–23, 36], making applicability of findings difficult. There is also a lack of large antenatal randomised controlled trials with maternal and foetal outcomes as the primary outcome [16, 17, 24].

## Methods

In the “REMOTE blood pressure monitoring in high-risk pregnant women — a randomised controlled trial” (REMOTE CONTROL trial), a pragmatic non-inferiority unblinded multicentre randomised controlled trial, we aim to determine whether remote BP monitoring in pregnant women at high risk for developing a HDP is as safe as conventional clinic BP monitoring. We will evaluate foetal outcomes as the primary outcome, in addition to maternal outcomes, patient satisfaction and the cost-effectiveness of this strategy. This study protocol follows the SPIRIT reporting guidelines [37].

### Design and setting

The REMOTE Control trial will recruit patients across 3 metropolitan Australian teaching hospitals; Liverpool, Campbelltown and Bankstown Hospitals. Within these hospitals, women at high risk of developing a hypertensive disorder of pregnancy are referred to specialist obstetric medicine clinics, where their blood pressure is monitored regularly throughout their pregnancy, leading to an additional 6–8 clinic reviews. The non-inferiority trial will compare remote blood pressure monitoring with conventional clinic monitoring in a 1:1 allocation ratio.

The trial has been prospectively registered with the Australian and New Zealand Clinical Trials Registry (ACTRN12620001049965p) in October 2020. Ethics approval was granted by the South Western Sydney Local Health District human research ethics committee (SWSLHD HREC) in June 2021.

### Study population

The study population will be all pregnant women at high risk for developing a hypertensive disorder of pregnancy, that attend the hospital sites for antenatal care. Eligible women must be ≥ 18 years old with at least one of the following high risk features: (1) chronic hypertension; (2) autoimmune disease; (3) pre-existing diabetes mellitus; (4) chronic kidney disease; (5) previous hypertensive disorder of pregnancy; (6) diagnosed with gestational hypertension; (7) IVF pregnancy; or (8) women whose risk of developing early-onset preeclampsia on first-trimester screening is reported to be < 1:100 [38]. Exclusion criteria for participation in the study are (1) evidence of preeclampsia on initial evaluation; (2) inability to access or use a smartphone as assessed by the research midwife on screening and consent; (3) inability to consent or (4) insufficient knowledge of English or other language supported by the application; (5) multiple pregnancy; and (6) lethal foetal abnormalities. The full inclusion criteria, exclusion criteria and definitions are listed in Table 1.

**Table 1:** REMOTE CONTROL trial inclusion and exclusion criteria

	**Definition**
**Inclusion criteria**	
Age ≥ 18 years	
Able to provide informed consent	
Able and willing to follow instructions for use of automated BP machine and app-based system	
Chronic kidney disease	Abnormalities of kidney structure or function (either reduced GFR < 90 ml/min/1.73 m^2^, albuminuria or proteinuria) present prior to pregnancy
Autoimmune disorder	Diagnosis of an autoimmune condition such as systematic lupus erythematosus prior to pregnancy
Type 1 or type 2 diabetes mellitus	
Chronic hypertension	Systolic BP > 140 mmHg or diastolic BP > 90 mmHg on at least 2 occasions prior to 20 weeks’ gestation
Previous pregnancy complicated by HDP or diagnosis of gestational hypertension this pregnancy	Previous HDP includes chronic hypertension, gestational hypertension, preeclampsia, or preeclampsia superimposed on chronic hypertension Gestational hypertension (Systolic BP > 140 mmHg or diastolic BP > 90 mmHg on at least 2 occasions after 20 weeks’ gestation)
Confirmation of viable intrauterine pregnancy on dating scan	
IVF pregnancy	
High-risk for developing preeclampsia on first-trimester screening	Reported risk of < 1:100 of developing preeclampsia on commercially available combined first-trimester screening tests
Before 28 weeks gestation	
**Exclusion criteria**	
Participants who have signs of preeclampsia on initial evaluation	Preeclampsia (BP reading > 140 mmHg and/or 90 mmHg on at least 2 occasions after 20 weeks’ gestation accompanied by evidence of end-organ involvement)
Multifoetal pregnancy	
Lethal foetal abnormality	Lethal foetal chromosomal, genetic or morphological abnormality detected on screening tests
Inability to access or use the app-based system	
Unable to consent	
Non-English speaking background not supported by the mobile application	

*GFR* glomerular filtration rate, *SLE* systemic lupus erythematosus, *BP* blood pressure, *HDP* hypertensive disorder of pregnancy

**Fig. 1:**
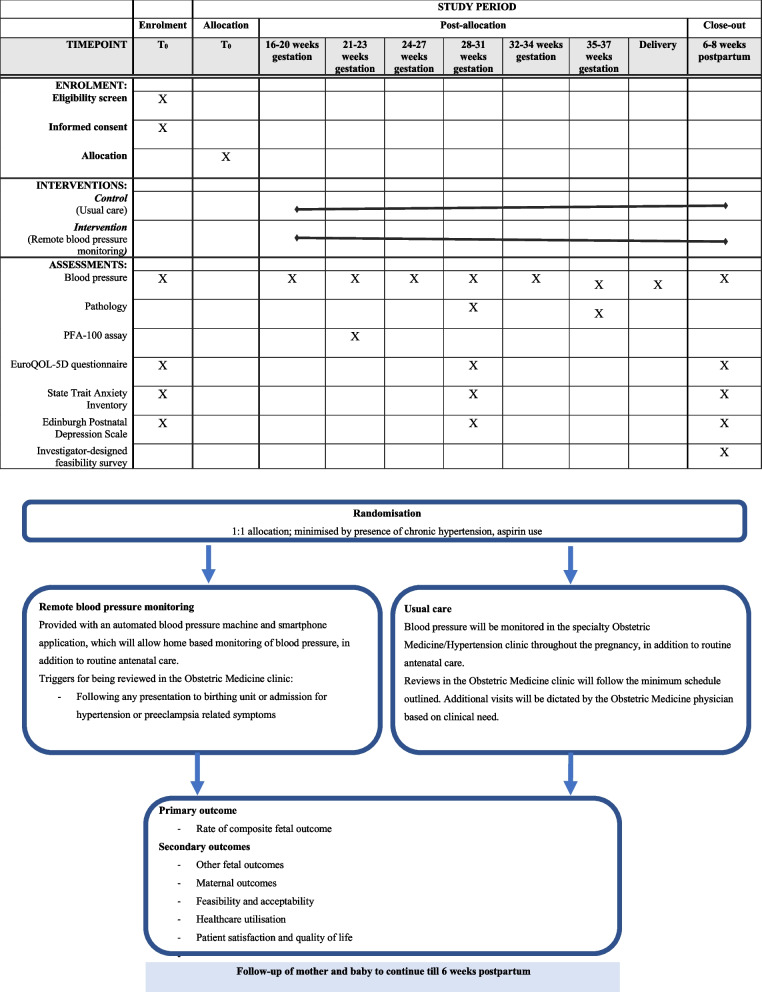
Schedule of enrolment, interventions, assessments and overview of study flow

Participants will be recruited through both the antenatal and specialist obstetric medicine clinics. Eligible patients will undertake full informed written consent with an Obstetric Physician or research midwife. Consented participants will be randomised in a 1:1 ratio, to receive either conventional care with face-to-face consultations or remote BP monitoring. Computer-generated randomisation will occur through RedCAP™, and a minimisation protocol for the variables chronic hypertension, centre of treatment and aspirin use will be instituted. Individual patient randomisation will be used so that treatment allocation remains unpredictable. To avoid bias, the random allocation sequence is concealed from those responsible for recruiting women into the study, and generated by the REDCap randomisation module. A research midwife based at each site will be responsible for consenting and assigning participants to the trial arms. Blinding of the treatment allocation to the patient and treating physician will be difficult and unethical. Outcome assessors and data analysts will not be blinded to treatment allocation. An overview of the study procedures is shown in Fig. 1.

#### Intervention arm: remote monitoring of BP

Prior to the start of the study, we will provide training of the remote monitoring strategy at each site to ensure familiarity with the technological aspects. A research midwife at each centre will be trained how to register, train and enrol new participants on the novel platform (consisting of an automated blood pressure device connected via Bluetooth to a smartphone application) after randomisation. As set in the research protocol, responsibilities of the clinicians will include review of uploaded parameters, management after reviewing new results and telephone contact with the pregnant women at home.

Women randomised to remote monitoring will be provided with an automated BP machine validated for use in pregnancy and preeclampsia (iHealth Track), which uploads blood pressure readings to a smartphone application (M♡THer). They will be educated on its use at their first visit by a research midwife or investigator. The smartphone application and innovative platform (M♡THer), was developed by the Commonwealth Scientific and Industrial Research Organisation (CSIRO) Australian e-Health Research Centre (AEHRC) and has been found to be feasible in supporting remote monitoring of women with diagnosis of gestational diabetes [39]. For this trial the application has been customised to focus on BP monitoring.

Education will be provided through a standardised video created by the trial team in addition to a patient information sheet with telephone contact numbers for technical or health-related questions. Participants will have any questions about the intervention answered.

Participants will be asked to take 3 readings, 5 min apart while sitting quietly at any time of the day. The first reading will be disregarded, and the higher of the last two readings will be recorded. This will occur on 3 occasions every week. The blood pressure reading, in addition to a symptom check-list through the M♡THer application, will be uploaded to a secure web-based dashboard. A pre-specified evidence-based algorithm built-in to the M♡THer application (Table 2), consistent with NICE guidelines for BP targets in pregnancy [2], will provide instructions to the patient. Patient data uploaded to the dashboard will be reviewed three times a week by an obstetric medicine clinician. Possible steps in the management, after the uploaded results are checked are (1) expectant management, (2) titration of medications by telephone call, (3) participant booked to attend a Telehealth appointment with the Obstetric Medicine clinic, (4) same-day clinical assessment or (5) hospital admission. Should hospital admission be necessary due to clinical deterioration, the patient will be monitored in the hospital as per local protocol and all relevant data will be collected. If discharged, women will continue remote BP monitoring until 6 weeks postpartum. All events of healthcare utilisation including imaging, pathology tests, consultations in the outpatient department and community-based care as well as ward admissions will be recorded.

**Table 2:**
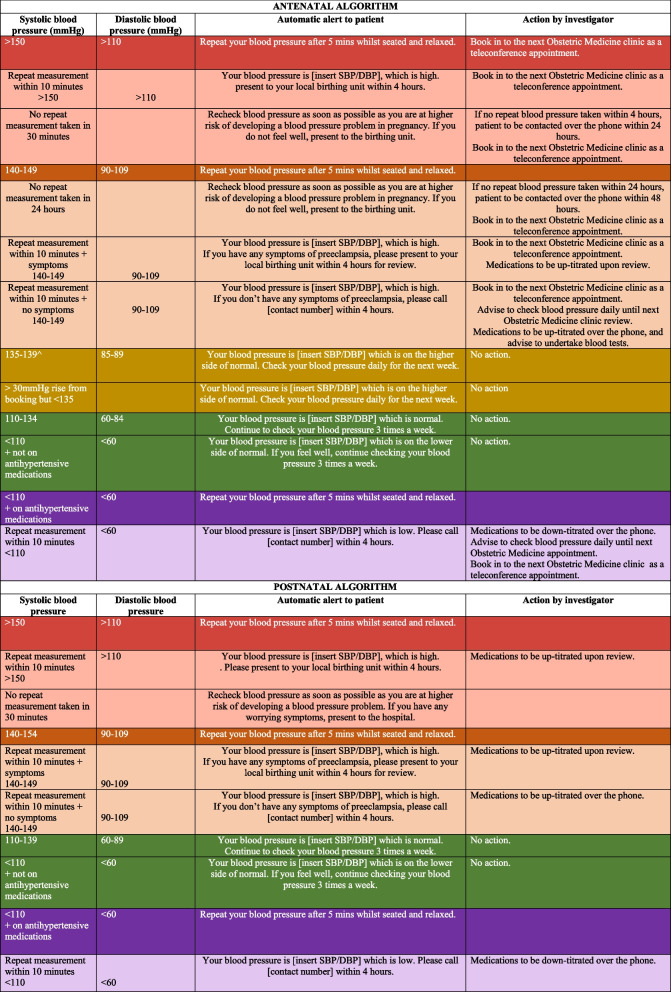
In-built prespecified blood pressure algorithms

#### Control arm: usual care

**Fig. 2:**
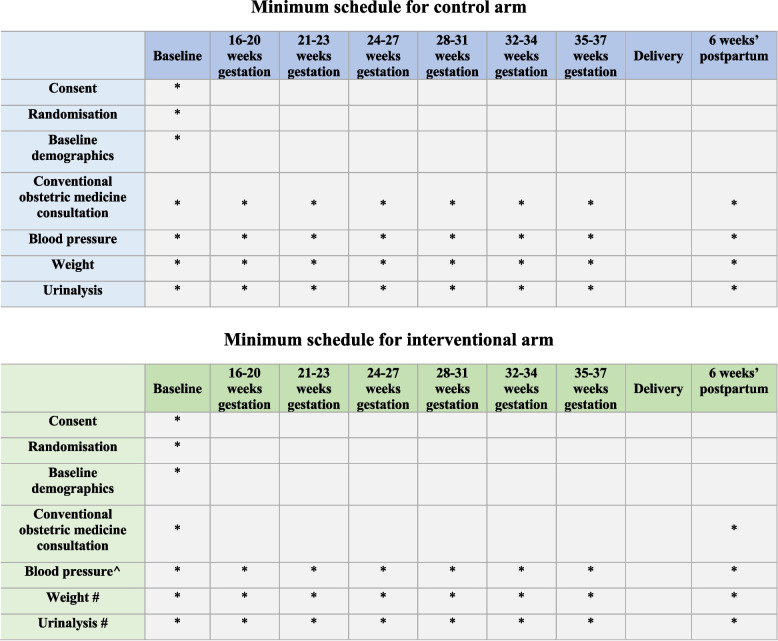
Participant timeline

Women in the control arm will have their blood pressure monitored in face-to-face consultations, and initiation and/or adjustment of medication (where appropriate) based on these measurements at the discretion of the Obstetric or Obstetric Medicine physician. The frequency of these specialty appointments will be dictated by clinical need as well as local current practice, but will follow the minimum schedule of at least 6 outpatient appointments, in addition to the routine antenatal schedule (Fig. 2). Women will return to the clinic for follow-up at 6 (+ / − 2) weeks postpartum.

Increasingly, women are utilising home blood pressure monitoring during pregnancy. However, the interpretation of these blood pressure readings is left to the patient, without clear guidance on normal and abnormal values in pregnancy. Our trial aims to evaluate the effectiveness and safety of remote blood pressure monitoring. Remote blood pressure monitoring in this trial refers to not only home blood pressure monitoring, but also a system whereby readings are transmitted to clinicians for review, in addition to immediate interpretation for patients about normal and abnormal values in the context of pregnancy, founded on evidence-based guidelines. This allows for proactive, rather than reactive care.

Therefore, as a pragmatic trial, women within the control arm may still undertake home blood pressure monitoring but are not encouraged to do so. This would fall within the scope of conventional care as defined in the trial protocol. For participants within the usual care arm who undertake home blood pressure monitoring using their own devices, decisions about clinical care will not be made based on home blood pressure readings provided by the patient in between appointments. However, for participants who report hypertension measured at home, usual clinical practice would be followed. Clinicians would validate this through more regular clinical visits, undertaking appropriate investigations and a formal blood pressure assessment in the fetomaternal assessment unit. Participants will also be advised that should they have clinical concerns, they should present to their local birthing unit for assessment.

Standard of care with regards to all other aspects of their antenatal care will be otherwise instituted for both groups including early initiation of low dose aspirin (LDA). Compliance with LDA will be assessed through obtaining a platelet function analyser (PFA-100) assay between 20 and 23 weeks’ gestation [4]. A routine set of pathology including full blood count, renal function testing, liver enzymes and maternal protein-creatinine ratio will be undertaken at 28 weeks’ and 36 weeks’ gestation. All women will be reminded to report, between and at routine antenatal visits, any new symptoms consistent with possible pre-eclampsia. If there are concerns regarding the development of preeclampsia, a minimum set of investigations will include a full blood count, renal function testing, liver enzymes, a maternal urine protein-creatinine ratio and soluble fms-like tyrosine kinase-1 to placental growth factor ratio (sFLT-1/PlGF ratio). Foetal assessment will be undertaken by measurement of foetal heart rate and pattern using a cardiotocography (CTG) and, if indicated, detailed ultrasonographic assessment. The remainder of antenatal care, in particular the timing and mode of delivery will be undertaken as per local practice. No concomitant treatments are prohibited during the trial.

### Outcome measures

The primary outcome is perinatal mortality and morbidity, represented by a composite adverse foetal outcome. The composite adverse foetal outcome is defined as: perinatal loss (miscarriage, pregnancy termination, stillbirth or neonatal death), high-level neonatal care (admission to neonatal intensive care unit or special care nursery for > 48 h) or small-for-gestational age (birthweight below the 10th centile). All components of the composite outcome will be assessed separately individually as secondary outcomes. Other secondary outcomes will consist of maternal outcomes, feasibility and patient satisfaction, quality of life, healthcare utilisation and cost-effectiveness (Table 3).

**Table 3:** Secondary outcomes

*General*
Rate of composite maternal complications (adapted from the International Collaboration to Harmonise Outcomes for Pre-eclampsia [40]: - Maternal mortality - Eclampsia - Stroke -Cortical blindness - Retinal detachment - Pulmonary oedema - Acute kidney injury - Liver capsule haematoma or rupture - Placental abruption - Postpartum haemorrhage - Raised liver enzymes - Haemolysis - Low platelets - Admission to the intensive care unit - Intubation/mechanical ventilation
Gestational age at delivery
Birthweight
APGAR scores at 1 min and 5 min
Neonatal seizures
Neonatal respiratory morbidity
Uncontrolled hypertension
Pregnancy prolongation
Rate of preeclampsia
Rate of induction of labour
Mode of delivery
Postnatal readmission rate
Second trimester mean systolic and diastolic BP
Third trimester mean systolic and diastolic BP
Postnatal mean systolic and diastolic BP
Mean systolic and diastolic BP obtained in clinic setting
Area under the BP curve
Proportion of readings within target (< 140/90 mmHg)
Episodes of severe hypertension > 160/100 mmHg
Time to medication institution (weeks’ gestation)
Number of medication dose escalations
***Healthcare utilisation***
Maternal blood or urine testing at the laboratory prior to birth admission
Foetal cardiotocography
Foetal ultrasound
Number of outpatient visits - General practitioner - Antenatal clinic visit - Obstetric medicine clinic visits
Number of medical, day, or maternity assessment unit visits
Acute care area for urgent/emergent visit other than in labour - Emergency department - Birthing unit presentations
Number of antenatal admission days prior to birth
***Feasibility and acceptability***
Compliance with remote monitoring *(interventional arm only)*
Compliance with face to face consultations
Number of phone calls from the investigator *(interventional arm only)*
Recruitment rate
Persistence with remote monitoring
***Quality of life and patient satisfaction***
EuroQol 5D survey
State Trait Anxiety Inventory questionnaire
Edinburgh Postnatal Depression Score questionnaire
Investigator-designed remote monitoring feasibility surveys (*interventional arm only*)
Qualitative interview with participants and clinicians *(interventional arm only)*

The satisfaction and quality of life of every participant will be surveyed with the EuroQol-5D-3L [41], State Trait Anxiety Inventory [42] and Edinburgh Postnatal Depression Score questionnaires [43]. Surveys will be sent by email at study baseline, at 28 weeks’ gestation and 6 weeks after delivery. Additionally, participants in the interventional arm will complete investigator-designed surveys to assess the technological feasibility of remote monitoring.

Qualitative interviews will be undertaken after the endpoint of the study using a mixed-methods approach, focusing on the experiences of clinicians and midwives involved in the study, as well as a group of consented participants from the interventional arm. Interviews will be conducted either in person or through virtual video conference, by an investigator not previously known to the participants during their pregnancy. All audio from the interviews will be recorded to allow for transcription, and numbers of participant to be determined by assessment of data saturation.

Australia has a universal healthcare system and as such Medicare is a national system that funds and also collects accurate data regarding episodes of health care delivery in the community including investigations, admissions, primary health review and medication prescription. Therefore, utilisation of healthcare resources will be assessed through collection of data from the Medicare Benefits Schedule (MBS) and Pharmaceutical Benefits Schedule (PBS). The cost-effectiveness of remote blood pressure monitoring will be assessed from the perspective of both patients and the healthcare system based on this data.

### Sample size

The sample size calculation is based on the assumption that the composite foetal outcome will be equal in the remote BP monitoring and control groups: a non-inferiority trial. Prior data indicates that the rate of the composite foetal outcome is approximately 31.1%, drawn from a study previously undertaken in the local high-risk pregnant population [4]. Investigators made a reasoned choice about the acceptable difference in adverse foetal outcome and feasibility of the trial. As a result, the non-inferiority margin was defined as a 15% absolute increase or less in the remote BP monitoring group. With a one-sided α of 0.05, the study will achieve a power of 0.80 if 118 women will be included in each trial arm. Furthermore allowing for a 20% attrition rate, 260 participants will be needed in total, 130 participants in each arm.

### Data handling, analysis and result reporting

At study entry, baseline demographics, medical and obstetric history will be collected. Antenatal data pertaining to the secondary outcomes will be collected throughout gestation including blood pressure data, investigations undertaken and any contact with the healthcare system. For the interventional arm, the M♡THer application will collect blood pressure data, symptom checklists and weight. At delivery, relevant data will be collected from the electronic medical record for the assessment of foetal outcomes such as gestational age at birth, birth weight, Apgar scores, and high-level neonatal care admission. For maternal outcomes, data will be collected from the electronic medical record on mode of delivery, antihypertensive medication use and adverse outcomes. Standardised online case report forms developed by investigators through REDCap™ will be used, and data will be stored on password-protected cloud drives located behind a university firewall, available only to investigators.

Data analyses will primarily be carried out according to the intention-to-treat principle but we will also perform per protocol analyses excluding participants in whom there is a clear deviation of the intended care as prescribed by the protocol in either the control group or the remote monitoring group. We will undertake both analyses with and without imputed data. We anticipate that some patients within the conservative care arm will undertake home blood pressure monitoring using their own devices. In such cases, we will ensure that during consultations, changes to medications will not be made based on home blood pressure readings provided by the patient. However, if there are clinical concerns, we would advise participants in the conventional arm to be assessed in the local birthing unit as is current standard of care. The case reporting form will take note of patients who are part of the conservative care arm that own blood pressure machines, and this will be adjusted for in a sensitivity analysis as a potential confounder.

Results will be reported according to Consolidated Standards of Reporting Trials (CONSORT) guidelines, using the extension for non-inferiority trials [44].

The dichotomous primary outcome will be analysed using a generalised linear model (with binomial distribution and log link), adjusting for minimisation variables, chronic hypertension and aspirin use. Treatment effects will be expressed as adjusted risk ratios with 95% CIs. The secondary outcomes that are binary will be analysed using the same methods described for the primary outcomes, with corresponding 95% CIs. For those secondary outcomes that are continuous, data that is normally distributed will be analysed with parametric tests and non-normally distributed data will be analysed with non-parametric tests. Significance will be set at 0.05 and adjustments for multiple comparisons will be made where comparing several groups.

Subgroup analyses will be undertaken on (i) variables used in the minimisation algorithm (i.e. hypertension type [chronic or gestational hypertension] and LDA use); and (ii) other variables of prognostic significance pre-specified as ethnicity, body mass index, prior severe hypertension in the index pregnancy, antihypertensive therapy at randomisation, gestational diabetes mellitus at randomisation, and smoking status at randomisation. Subgroup analyses will be limited to the primary outcome. Results will be presented as adjusted risk ratios with 95% confidence intervals. The results of subgroup analyses will be treated with caution and will be used for the purposes of hypothesis generation only.

Participants with missing primary outcome data will not be included in the primary analysis in the first instance. This presents a risk of bias, and sensitivity analyses will be undertaken to assess the possible impact of the risk. Sensitivity analyses will also be undertaken for the women who were randomised to the conventional care arm, who had a remote blood pressure monitor at home. A post-hoc analysis will be undertaken for aspirin compliance as a modifier based on PFA-100 levels.

Comparison of questionnaires will be made for each time point.

A’within trial’ economic evaluation will be conducted alongside the trial from a health sector perspective, and extending to patient and carer costs. This will combine three forms of complimentary economic analysis in a comprehensive approach.

First, a cost effectiveness analysis (CEA) will compare remote monitoring against usual care and estimate the incremental cost per change in the primary composite adverse patient outcome. In the non-inferiority approach, it is hypothesized that remote monitoring will reduce costs with no significant change in outcomes. Costing will consider the difference in implementing the model of care between trial arms. In usual care, this will include the time cost of each specialist visit, and the time and travel cost of patients (and carers, if relevant). In remote monitoring, this will include the cost of each unit of equipment, development of training, and time in undertaking educational training (professional and patient), specialist time to review BP readings, and patient visit to specialists (similar to usual care, describe above). MBS will be used to cost professional time and average hourly wage rates used to value for patient/carer time. For both trial arms, health care utilisation will be compared and costed using reference costs from the Independent Hospital Pricing Authority (IHPA).

Secondly, a cost-utility analysis (CUA) will compare trial arms and estimate the incremental cost per change in ‘health utility’ as the outcome measure, replacing the composite adverse outcome. Health utility is an indexed measure of (preference-weighted) patient quality of life, will be measured by the EQ5D and used to generate quality-adjusted life years (QALYs). QALYs will then be converted to dollar values by multiplying each QALY by $50,000. By combining monetized QALYs and costs a measure of ‘net benefit’ is generated where a positive value indicates value for money.

Thirdly, a value of information (VOI) analysis will then estimate statistical uncertainty whether remote monitoring is cost-effective. In a Bayesian analysis, an estimate of the economic value of undertaking further research will be made to reduce parameter uncertainty (cost and outcomes) and increase confidence that remote monitoring is cost-effective. The VOI will then extend to investigate subgroup heterogeneity and to explore whether remote monitoring is relatively more (or less) cost-effective in particular groups, to inform whether further research can best focus on subgroups where uncertainty is greatest.

All adverse events experienced by the trial participant, from randomisation until 3 months postpartum, will be collected and considered for causal links to the study. Serious adverse events not pre-specified as protocol-exempt will be reported to the data monitoring committee. The data monitoring committee will consist of an Obstetrician and Obstetric Medicine physician, independent from the sponsor and competing interests. The data monitoring committee and ethics committee will meet to review trial conduct if adverse events arise during the trial. The Trial Management Group (TMG) will include those individuals responsible for the day-to-day management of the trial — the coordinating principal investigator (CPI) and the site lead investigators and site allocated research midwife. The role of the group is to monitor all aspects of the conduct and progress of the trial, ensure that the protocol is adhered to and take appropriate action to safeguard participants and the quality of the trial itself. The TMG will meet 3-monthly. No problems directly related to the intervention that are detrimental to the participants are anticipated, and therefore no interim analyses and formal stopping guidelines have been outlined.

### Ethics and dissemination

Changes to the study protocol are documented in amendments and submitted for approval to the SWSLHD HREC. After completion of the trial, the principal investigator will report on the results of the main study and submit a manuscript to a peer-reviewed medical journal. Supplementary analyses will be reported separately. On study completion, data will be made available upon request.

## Discussion

The REMOTE CONTROL trial will be the first non-inferiority randomised controlled trial, powered to evaluate maternal and foetal outcomes conducted within the Australian healthcare system. A composite foetal events outcome was chosen as the primary outcome, as this represents a definitive end-point relevant to clinicians, patients and the healthcare system. If foetal and secondary outcomes from this study are similar with the institution of remote monitoring, there are major potential benefits to patients and the healthcare system. Through reducing the travel costs, clinic visits and waiting times, it is likely to lead to improved compliance for all patients. In addition, it could improve delivery of care to vulnerable populations in rural and remote communities in Australia, which has unique geographic and social considerations.

The trial is designed to be a pragmatic non-inferiority randomised controlled trial. The underlying pathophysiology of HDP is not going to be altered by the proposed intervention, and therefore a non-inferiority trial design was opted for.

The participant population will be those deemed to be high risk for developing a hypertensive disorder of pregnancy. It is this group of women who are currently being referred to and monitored in an Obstetric Medicine clinic within our local health district. This leads to additional visits throughout their pregnancy, in addition to routine antenatal care. In this high-risk population, the rate of HDP in a recent study set in South Western Sydney was 33.8%, with the rate of adverse foetal outcomes (intrauterine growth restriction or pre-term delivery) between approximately 15–17% (4). Therefore, the high incidence of HDP and foetal adverse outcomes in this population lends itself to an adequately powered study without requiring overwhelming numbers to detect a difference between the groups.

### Trial status

Recruitment commenced in July 2022, with planned end date of the trial to be September 2023. The protocol is version 2 dated 28th April 2021.

## Data Availability

Full trial dataset will be available to the Coordinating Principal Investigator and site lead investigators. The CSIRO AEHRC team will only be able to access de-identified, grouped data so that they are able to audit the processes, use and feasibility of their application. They will not be involved in the data collection of the study. Deidentified research data will be made publicly available when the study is completed, published, upon ethics approval. The author’s will need to be approached for data to ensure it is appropriately handled. Statistical analysis will occur with commercially available statistical programs, SPSS (version 27).

## Abbreviations

AEHRC: Australian e-Health Research Centre
BP: Blood pressure
CEA: Cost effectiveness analysis
CONSORT: Consolidated Standards of Reporting Trials
CSIRO: Commonwealth Scientific and Industrial Research Organisation
CUA: Cost utility analysis
HDP: Hypertensive disorders of pregnancy
IHPA: Independent Hospital Pricing Authority
IVF: In vitro fertilisation
LDA: Low dose aspirin
MBS: Medicare benefits schedule
mHealth: Mobile health
NICE: National Institute for Health and Care Excellence
PBS: Pharmaceutical benefits schedule
PFA-100: Platelet function analyser-100
QALY: Quality-adjusted life years
SWSLHD HREC: South Western Sydney Local Health District Human Research Ethics Committee
TMG: Trial Management Group
VOI: Value of information
